# A Textbook of Histology

**Published:** 1907-03

**Authors:** 


**A Textbook of Histology.** By **Frederick R. Bailey,** M.D., Adjunct Professor of Normal Histology, Medical Department of Columbia University, New York. Second, revised edition. Illustrated. Octavo, pp. 497. New York: William Wood & Co. 1906. (Price, $3.00).

The general excellence of the first edition of this work has been materially improved in the present volume by the betterment of some of the drawings and the addition of new plates. The chapter on the nervous system for which the author apologised in the first edition has proven to be of great value and has been amplified here, there being more noticeable changes in this section than in any other part of the book. It has been made necessary by the advances in neurohistology. To add to the completeness of the chapter several new diagrams have been added. The primary aim of the writer was to prepare a textbook which would be of assistance to the student in the laboratory and a genuine aid to the instructor, and he has admirably succeeded. Most excellent are the chapters on the urinary system and the reproductive organs. The illustrations and press work are beyond criticism.

